# Association of cigarette smoking with cardiovascular risks and quality of life in patients with schizophrenia: a cross-sectional study

**DOI:** 10.1192/j.eurpsy.2026.12156

**Published:** 2026-07-31

**Authors:** P. K. Lee, X. Zhang, Y. W. Mak

**Affiliations:** 1 Department of Health Technology and Informatics; 2 School of Nursing, The Hong Kong Polytechnic University, Hong Kong, China

## Abstract

**Introduction:**

People with schizophrenia have poorer physical health than the general population due to poor diet, lack of physical activities, and high rates of obesity. This population has been reported to have lower quality of life across physical, psychological, and social health. Furthermore, these patients have a higher prevalence of smoking and lower quit rates when compared to general populations. Although smoking increases the risk of cardiovascular events, which are associated with an increased risk of premature death, limited research has compared cardiovascular risk and quality of life across different smoking statuses.

**Objectives:**

This study aimed to determine demographic and clinical differences in schizophrenia patients, including cardiovascular risks and health-related quality of life, by smoking status.

**Methods:**

Baseline data were extracted from a longitudinal study. The primary objective of the study was to investigate the effects of different smoking cessation therapies using fMRI. Between 2022-2024, participants were recruited from local ICCMW centers and psychiatric outpatient clinics in Hong Kong. All participants were patients diagnosed with schizophrenia for more than one year and stable on medication. Smokers were required to agree to quit smoking and not have other substance use disorders. Participants were divided into two groups based on smoking status: never smokers and ever smokers (current and former). Information about cardiovascular risks (including BMI, body fat percentage, blood pressure, blood oxygen saturation, carbon monoxide level, clinical history) was collected. The EQ-5D5L was used to measure quality of life. To identify possible predictors for non-participation, a multivariate regression model was used.

**Results:**

There are 55 never smokers (57.9%) and 40 ever smokers (42.1%) included in the study (N = 95). Significant demographic difference was found in gender (p = 0.022), marital status (p = 0.020), education attainment (p = 0.002), and type of housing (p = 0.002). In terms of clinical differences, BMI and CO level were significantly different between the two groups, with p = 0.022 and p = 0.002 respectively. No significant correlation was found for other indicators, including body fat percentage, systolic blood pressure, diastolic blood pressure, blood oxygen saturation, history of high cholesterol, hypertension, diabetes, and quality of life. After regression analysis in participants and non-participants, the adjusted odds ratios for education level, employment status, and type of housing were 1.63 [95% CI: 0.747-3.559], 2904617146.5 [0], and 0.377 [0.178-0.802] respectively.

**Conclusions:**

The results show that ever-smoking individuals with schizophrenia may have higher cardiovascular risks in some areas. These results should be interpreted with caution due to sampling and self-reporting biases.

**Disclosure of Interest:**

None Declared

